# Vascular disrupting agent for neovascular age related macular degeneration: a pilot study of the safety and efficacy of intravenous combretastatin a-4 phosphate

**DOI:** 10.1186/2050-6511-14-7

**Published:** 2013-01-14

**Authors:** Mohamed A Ibrahim, Diana V Do, Yasir J Sepah, Syed M Shah, Elizabeth Van Anden, Gulnar Hafiz, J Kevin Donahue, Richard Rivers, Jai Balkissoon, James T Handa, Peter A Campochiaro, Quan Dong Nguyen

**Affiliations:** 1 Wilmer Eye Institute, Johns Hopkins University School of Medicine, 600 North Wolfe Street, Maumenee 745, Baltimore, MD 21287, USA; 2 Division of Cardiology, Department of Medicine, Johns Hopkins University School of Medicine, Baltimore, MD, USA; 3 Department of Medicine, Case Western Reserve University School of Medicine, Cleveland, OH, USA; 4 Department of Anesthesia, Johns Hopkins University School of Medicine, Baltimore, MD, USA; 5 OxiGene, Inc., South San Francisco, California, CA, USA; 6 Stanley M. Truhlsen Eye Institute, University of Nebraska Medical Center, Omaha, NE, USA

**Keywords:** Angiogenesis, Neovascularization, Ocular pharmacology, Retinal degeneration, Combretastatin A-4 Phosphate, CA4P, Vascular disrupting agents, VDA

## Abstract

**Background:**

This study was designed to assess the safety, tolerability, and efficacy of intravenous infusion of CA4P in patients with neovascular age-related macular degeneration (AMD).

**Methods:**

Prospective, interventional, dose-escalation clinical trial. Eight patients with neovascular AMD refractory to at least 2 sessions of photodynamic therapy received CA4P at a dose of 27 or 36 mg/m^2 ^as weekly intravenous infusion for 4 consecutive weeks. Safety was monitored by vital signs, ocular and physical examinations, electrocardiogram, routine laboratory tests, and collection of adverse events. Efficacy was assessed using retinal fluorescein angiography, optical coherence tomography, and best corrected visual acuity (BCVA).

**Results:**

The most common adverse events were elevated blood pressure (46.7%), QTc prolongation (23.3%), elevated temperature (13.3%), and headache (10%), followed by nausea and eye injection (6.7%). There were no adverse events that were considered severe in intensity and none resulted in discontinuation of treatment. There was reduction of the excess foveal thickness by 24.15% at end of treatment period and by 43.75% at end of the two-month follow-up (p = 0.674 and 0.161, respectively). BCVA remained stable throughout the treatment and follow-up periods.

**Conclusions:**

The safety profile of intravenous CA4P was consistent with that reported in oncology trials of CA4P and with the class effects of vascular disruptive agents; however, the frequency of adverse events was different. There are evidences to suggest potential efficacy of CA4P in neovascular AMD. However, the level of systemic safety and efficacy indicates that systemic CA4P may not be suitable as an alternative monotherapy to current standard-of-care therapy.

**Trial registration:**

ClinicalTrials.gov NCT01570790.

## Background

Combretastatin-A4-phosphate (CA4P) is a vascular disrupting agent (VDA), a class of experimental medications that lead to collapse or occlusion of abnormal vascular structures. CA4P is a synthetic phosphorylated pro-drug of CA4, a naturally occurring derivative of the South African willow tree, *combretum caffrum*, which reversibly binds tubulin at the colchicine-binding site to inhibit microtubule assembly. The mechanism by which CA4P and CA4 act on pathologic neovasculature is not completely understood; although it appears that through its reversible binding to tubulin, CA4P causes distortion and detachment of immature proliferating endothelial cells in abnormal vasculature (mature endothelial cell shape is maintained by the secondary scaffolding protein actin). Because of its reversible effects and the short half-life of about 10-27 min, as demonstrated in animal studies, CA4P does not display the side effects typical of tubulin binding inhibitors [1]. Although it is a vascular targeted agent, its specific mechanism of action and side effect profile differ from those of vascular endothelial growth factor (VEGF) inhibitors [2].

CA4P has been shown to disrupt tumor neovasculature and decrease tumor blood flow in both animals and humans [3]. Multiple human studies have demonstrated significant decrease in tumor blood flow within a few hours of CA4P administration, whether examined with dynamic contrast enhanced (DCE), MRI, PET scan or perfusion CT scan. The safety profile of CA4P in oncology patients suggests that adverse effects are generally mild to moderate and mostly occur in the few hours following an infusion. Consistent with its vascular activity, the most typical adverse effects in descending order are nausea, headache, tumor pain, fatigue, vomiting, sinus tachycardia and bradycardia, paresthesia, diarrhea, sweating, and transient hypertension and QTc prolongation [4].

CA4P was reported to decrease neovascularization in mice with laser-induced disruption of Bruch’s membrane, in VEGF overexpressing mice, and in a model of retinopathy of prematurity (ROP) induced by excessive oxygen [5,6]. In the ROP model, CA4P demonstrated specificity for abnormal vasculature with sparing of normal angiogenesis required to support growth of the developing eye. The dose of CA4P required in the laser burn model was approximately *30-fold* higher than that required in the other two models, suggesting greater potency on abnormal vascular structures in contrast to normal wound healing [6].

These preclinical observations along with the apparent tolerability of systemic CA4P in oncology patients [7-10] provided the rationale for the pilot study of intravenous CA4P in patients with neovascular AMD. The index study is the first clinical trial of CA4P in patients with an ophthalmic disorder, and is the first trial of systemic CA4P in a population of patients without malignancy. Therefore, the study may contribute unique safety and biological activity data to the VDA literature.

## Methods

### Selection criteria

A prospective, open-label, dose-escalation phase 1 study was conducted to assess the safety, tolerability, and potential efficacy of CA4P in patients with choroidal neovascularization (CNV) secondary to AMD. The clinical trial was approved by the Johns Hopkins Medicine Institutional Review Board (IRB). Prior to determination of eligibility for enrollment, patients provided informed written consent to participate in the Study and to allow the information about them such as their eye, gender, and age (but not their names and other specific identifiable information) to be published in scientific literature so that others can be educated and learn from the trial.

Fluorescein angiography (FA) was employed to document presence of active subfoveal CNV. Patients with conditions that might contribute to CNV, such as pathologic myopia, histoplasmosis, and others were excluded. The CNV lesion size was limited to ≤12 Macular Photocoagulation Study (MPS) disc areas, of which at least 50% had to be active CNV. Subretinal hemorrhage was limited to <50% of total lesion size and scarring or atrophy to <25% of lesion size.

All major types of CNV were eligible. However, in patients with minimally classic or purely occult CNV, there has to be a documented evidence of two or more lines of vision loss during the previous 12 weeks. Eyes with best corrected visual acuity (BCVA) equivalent to 20/40 or worse, as measured by ETDRS charts, were eligible provided BCVA in the fellow eye is equivalent to 20/800 or better. When both eyes were eligible, the eye with better vision was selected.

Patients with any therapy for neovascular AMD within 12 weeks of screening or with prior subfoveal thermal laser therapy were excluded. There were specific exclusions for history of hemorrhagic or bleeding disorders and for cardiac disease, including angina, myocardial infarction, congestive heart failure or diagnostic tests showing an ejection fraction less than 50%, atrial fibrillation, clinically significant arrhythmias, and syncope. Conditions or medications associated with QTc prolongation were excluded. A normal 12-lead electrocardiogram (ECG) showing a QTc < 440 ms was required within 4 weeks prior to enrollment. Patients were also required to have a normal cardiac stress test of any type within 2 months prior to study entry. Laboratory tests obtained prior to enrollment were required to demonstrate adequate bone marrow, hepatic and renal functions, and normal blood coagulation profile. Uncontrolled hypertension (defined as blood pressure consistently greater than 150/100 mmHg irrespective of medication) or uncontrolled hypokalemia unresponsive to supplementation and/or hypomagnesaemia were exclusion criteria.

### Treatment plan and study design

Patients received CA4P (OxiGene Inc., South San Francisco, CA) at a dose of 27 or 36 mg/m^2 ^as a 10-minute intravenous infusion weekly for 4 weeks. Vital signs were obtained every 15 minutes for two hours and then hourly for five hours after infusion. ECGs were collected hourly for five hours after completion of the infusion. Adverse events (AEs) were collected and graded using The NCI Common Terminology Criteria for Adverse Events (CTCAE) v 3.0. Subjects were reassessed and had to continue to meet inclusion/exclusion criteria for hematologic, hepatic, and renal functions prior to each scheduled dose. No further CA4P would be administered to any subjects who experienced AEs of Grade-2 or greater: ventricular arrhythmia, second or third degree AV block, severe sinus bradycardia less than 45 bpm or tachycardia >120 bpm not due to other causes (e.g. fever), persistent supraventricular arrhythmia *[*e.g. *atrial fibrillation, flutter, atrioventricular nodal tachycardia (AVNRT)]* lasting more than 24 hours, ventricular tachycardia defined as >9 beats in a row, or any length of torsades de pointes (polymorphic ventricular tachycardia with long QTc), or unexplained recurrent syncope), QTc prolongation in which the interval exceeds 500 msec on any two consecutive ECGs, Grade-2 or greater myocardial infarction, or ocular toxicities deemed by the investigator not acceptable for the patients to receive further treatments. A cardiac electrophysiologist (JKD) reviewed the ECGs and made recommendations pertaining to the conduct of the study. In addition, an anesthesiologist participated in taking care for the patients during the study, including the management of hypertension.

The study was designed as a single escalating dose with cohorts of five subjects. Escalation to the next cohort was based on the presence of no more than one subject with a dose limiting toxicity (DLT). DLTs were defined as specific events that are considered to be probably or definitely related to CA4P. Major DLTs included QTc interval ≥ 500 msec *(based on measurements provided by the core laboratory for ECG analysis),* Grade-2 or greater ventricular arrhythmia, unexplained syncope, Grade-3 or greater toxicity, delayed recovery postponing re-treatment by >14 days, and ocular toxicity such as keratopathy, uveitis, optic neuropathy, and retinopathy, at the discretion of the investigator.

Prior to each treatment, patients had ocular and physical examinations, ECG, complete blood count, and serum chemistry determinations. These safety tests were also performed 1 and 2 months following the last administration of CA4P. Ocular examination included slit-lamp biomicroscopy, indirect ophthalmoscopy, intraocular pressure (IOP) measurements, and BCVA. Assessment of CNV was performed using FA and OCT at screening, 1 hour after the first infusion, and immediately prior to the second, third, and fourth infusions, and at 4-week and 8-week visits. BCVA was assessed at all the visits prior to infusion.

#### Statistical methods

Descriptive statistical summaries were performed for safety and efficacy parameters. There were no predictive statistical designs used in this pilot study.

## Results

### Patient characteristics

Between August 2003 and May 2005, 15 patients with AMD were screened at a single center, the Wilmer Eye Institute at the Johns Hopkins University. Seven subjects were not eligible; eight subjects were enrolled; five subjects received 27 mg/m^2 ^and three subjects received 36 mg/m^2^. The age of the enrolled patients ranged from 57 to 84 years (Table 1). BCVA in the study eyes ranged from 25 to 73 letters and in the fellow eyes ranged from zero to 80 letters. At baseline, all patients had active subfoveal CNV: occult in 6 patients and minimally classic in two. In the fellow eyes, 5 patients had active CNV at baseline, one patient had history of CNV resolved with disciform scarring and light-perception vision, and two patients had no history of CNV. None of our patients was naïve to treatment at baseline with all of them receiving at least 2 sessions of photodynamic therapy (PDT) prior to the study (Table 1).

**Table 1:** Demographics of the study subjects who were treated with intravenous combretastatin A-4 phosphate and characteristics of study and fellow eyes at baseline

**Patient**	**Age**	**Gender**	**Race**	**Previous treatment**	**CNV SE**	**CNV FE**	**BCVA**		**FTH**	
							**SE**	**FE**	**SE**	**FE**
**1**	81	F	White	PDT × 3	Subfoveal occult	Disciform Scar*	60	25	187	127
**2**	82	F	Hispanic	PDT × 2	Subfoveal occult	Subfoveal occult disciform scar*	73	4	204	573
**3**	84	F	White	PDT × 2	Subfoveal occult	No CNV	64	80	404	183
**4**	57	M	White	PDT × 3	Subfoveal minimally classic	Extrafoveal active occult foveal disciform scar *	67	73	346	236
**5**	64	F	White	PDT × 4	Subfoveal occult with disciform scar	Disciform scar	30	PL	519	x
**6**	70	M	White	PDT × 2	Subfoveal minimally	Subfoveal occult*	72	47	366	256
**7**	75	F	White	PDT × 2	Subfoveal occult	No CNV	25	72	292	238
**8**	79	F	White	PDT × 3	Subfoveal occult	Subfoveal occult*	64	48	450	393
**Mean (±SD)**	74 (±9.6)						56.9 (18.7±)	49.9 (±27.9)	346.0 (±115.2)	286.6(±150.2)

The asterisks indicate the fellow eyes that had active CNV at baseline. *CNV* = Choroidal neovascular membrane; *SE* = Study Eye; *FE* = Fellow Eye, *BCVA* = Best corrected visual acuity; *FTH* = central foveal thickness; *PDT* = photodynamic therapy; *M* = Male; *F* = Female; *SD* = Standard deviation.

### Systemic safety

The majority of the adverse events were encountered during infusion and within the following 5 hours. The most common AEs were transient elevated blood pressure (46.7%), transient QTc prolongation (23.3%), elevated temperature (13.3%), and headache (10%), followed by nausea, and eye injection (6.7% each). Other noted AEs included T-wave inversion, tachycardia, premature ventricular contractions, and chest pain. All AEs resolved before dismissal of the patient no later than 5 hours post-infusion. There were no AEs that were considered serious or severe (Grade-3 or 4) and none resulted in discontinuation of treatment. One patient did not receive all four administrations of CA4P secondary to non-specific gastrointestinal symptoms that were not considered grade-3 or 4.

**Figure 1:**
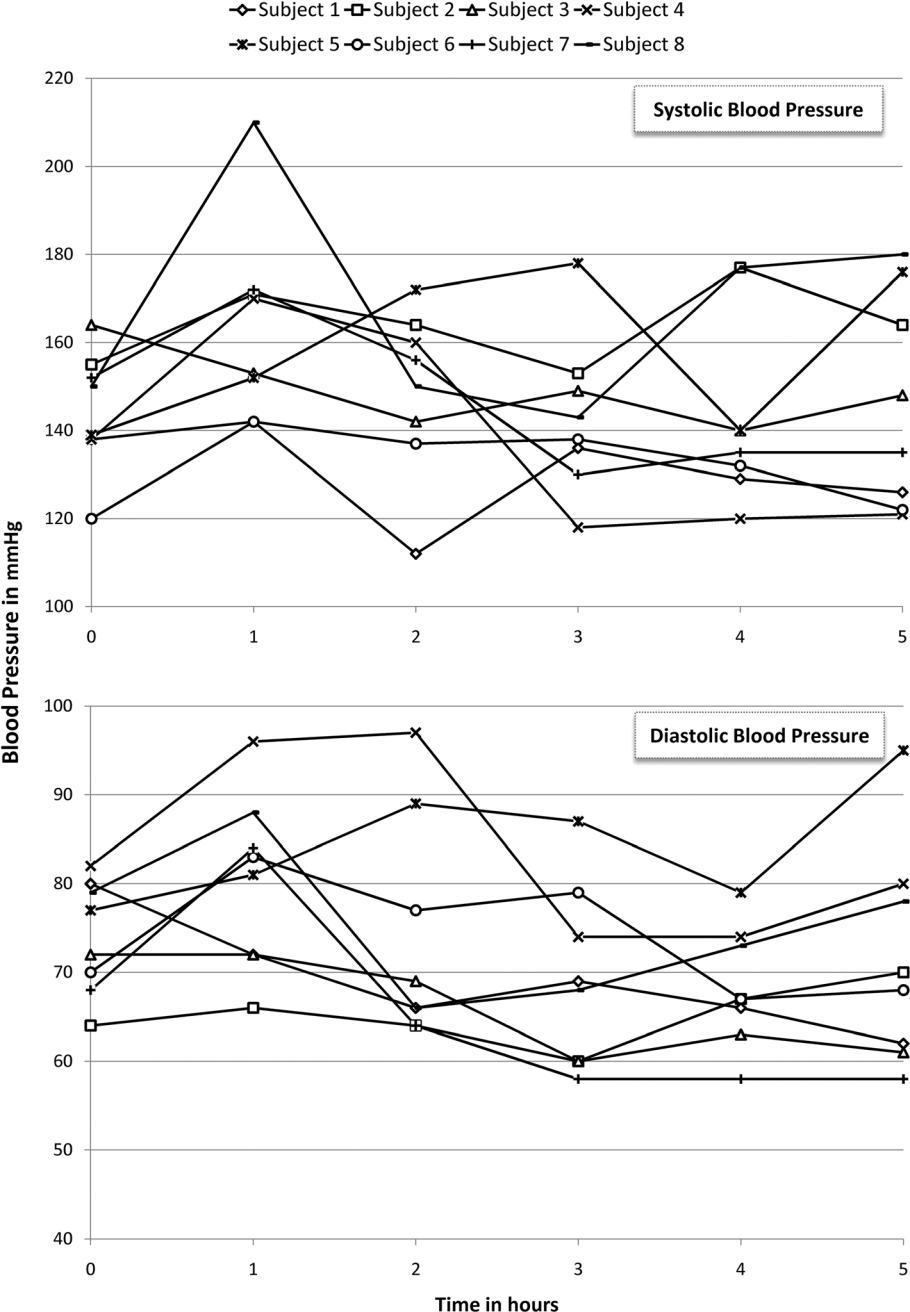
Changes in systolic (top) and diastolic (bottom) blood pressure in the study subjects following the first infusion of combretastatin A-4 phosphate.

Systolic and diastolic blood pressure changes following the first infusion of CA4P in each subject are shown in Figure 1. Prior to treatment, one subject was normotensive (systolic ≤120 mmHg), three were borderline hypertensive (systolic >120 and ≤140 mmHg), and four were hypertensive. The changes in the mean systolic blood pressure were similar following each of the four infusions (Table 2). The average systolic blood pressure at baseline was 145 mmHg and the average diastolic pressure was 74 mmHg. Six patients (75%) showed either worsening of the pre-existing systolic blood pressure (an increase of >20 mmHg) or development of hypertension (elevation of systolic blood pressure >140mgHg). The elevation in systolic blood pressure was transient and resolved spontaneously within the post-infusion period. Diastolic blood pressure did not show any significant change in any patient either after the infusion or during the course of the study. The average systolic blood pressure at the end of the study was 142 mmHg and the average diastolic blood pressure was 72 mmHg.

**Table 2:** Changes in the mean systolic blood pressure and QTc interval following each infusion of combretastatin A-4 phosphate (SE = Standard error)

	**1st infusion**		**2nd infusion**		**3rd infusion**		**4th infusion**	
	**mmHg *(SE)***	**QTc in μm *(SE)***	**mmHg *(SE)***	**QTc in μm *(SE)***	**mmHg *(SE)***	**QTc in μm *(SE)***	**mmHg *(SE)***	**QTc in μm *(SE)***
**Prior to Infusion**	145 *(4.8)*	411 *(3.51)*	131 *(3.38)*	406 *(3.25)*	136 *(4.78)*	412 *(3.58)*	132 *(7.11)*	415 *(6.14)*
**Hour 1**	164 *(7.9)*	419 *(4.41)*	165 *(5)*	405 *(4.75)*	163 *(9.48)*	416 *(3.44)*	154 *(4.84)*	417 *(4.82)*
**Hour 2**	149 *(6.66)*	430 *(10.12)*	148 *(5.53)*	427 *(8.09)*	148 *(5.32)*	428 *(6.62)*	139 *(6.56)*	425 *(8.75)*
**Hour 3**	143 *(6.31)*	424 *(7.23)*	141 *(2.95)*	422 *(4.78)*	139 *(5.33)*	428 *(6.01)*	136 *(7.8)*	424 *(7.47)*
**Hour 4**	144 *(7.6)*	426 *(4.93)*	137 *(4.5)*	426 *(4.78)*	131 *(5.75)*	424 *(5.09)*	128 *(6.63)*	423 *(7.35)*
**Hour 5**	148 *(9.7)*	423 *(7.55)*	137 *(5.08)*	429 *(12.83)*	142 *(3.62)*	423 *(6.61)*	138 *(5.72)*	421 *(7.65)*

One subject had a baseline pressure of 156/82 mmHg despite prescriptions of lisinopril, atenolol and hydrochlorothiazide. Following the first administration of CA4P, her blood pressure climbed to 216/102 mmHg 45 minutes post infusion and returned to baseline level two hours post-infusion. Prior to the planned second treatment, blood pressure was 176/84 mmHg, so CA4P was withheld and lisinopril dose was increased. Prior to next infusion, blood pressure was 164/75 mmHg and peaked to 226/102 mmHg one hour post-treatment. The subject complained of chest heaviness that was not accompanied by ECG changes. Two doses of nitroglycerine were administered without relief; however, an oral antacid relieved the symptoms. The absence of ECG changes and the resolution of heartburn with antacid/belching suggested the symptoms to be of gastro-intestinal origin. Due to the adverse experiences, the patient did not receive any further administration of CA4P.

**Figure 2:**
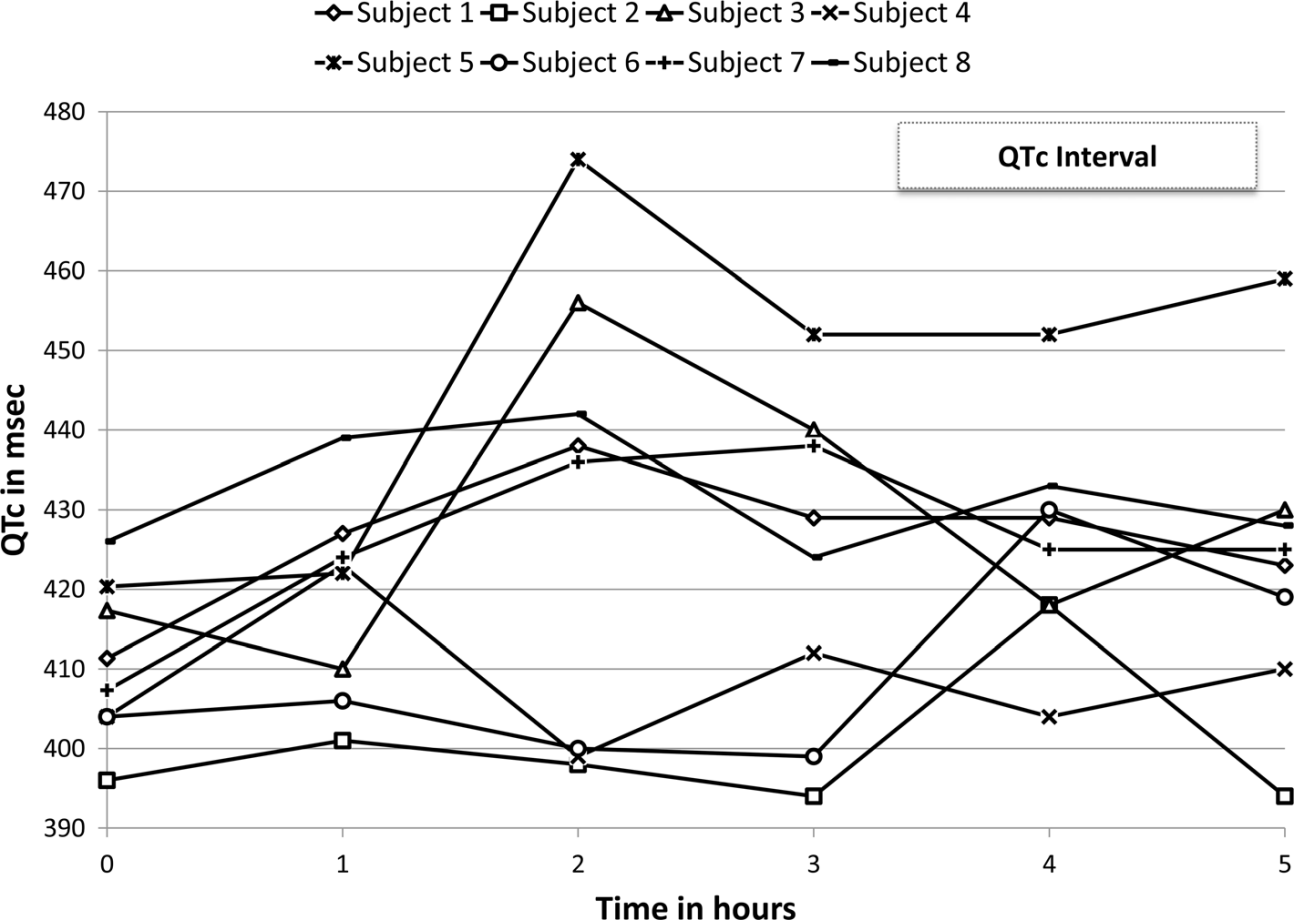
Changes in the QTc interval in the study subjects following the first infusion of combretastatin A-4 phosphate.

Baseline QTc was normal for all patients (median 409 ms, range 396-426 ms). QTc increased in all patients after infusion of CA4P, with a peak QTc significantly higher than baseline (median 438 ms, range 418–474 ms, p < 0.05). The changes in QTc interval following first infusion of CA4P are shown in Figure 2. The time-to-peak QTc was a median of 2 hours post-infusion (range 1–5 hours). Two female patients had grade-1 prolongation of QTc ≥450 ms *(3/4 infusions; patients 3 and 5)*, and one had a grade-2 complication of QTc peak ≥ 470 ms *(1/4 infusions: patient 5).* The relative increase in QTc did not correlate with baseline QTc *(linear regression r = 0.15, p = 0.49*). Mean changes in QTc are summarized in Table 2.

### Ocular safety

Two of the eight patients experienced unilateral conjunctival injection during one of their treatments; another patient reported flashes and floaters in one eye. Both observations were transient and resolved without sequelae. Both observations were judged to be related to the study drug but were not serious adverse events. Since no DLTs were observed, a maximal tolerated dose (MTD) was not defined in this study.

### Ocular efficacy

#### Study eyes

**Figure 3:**
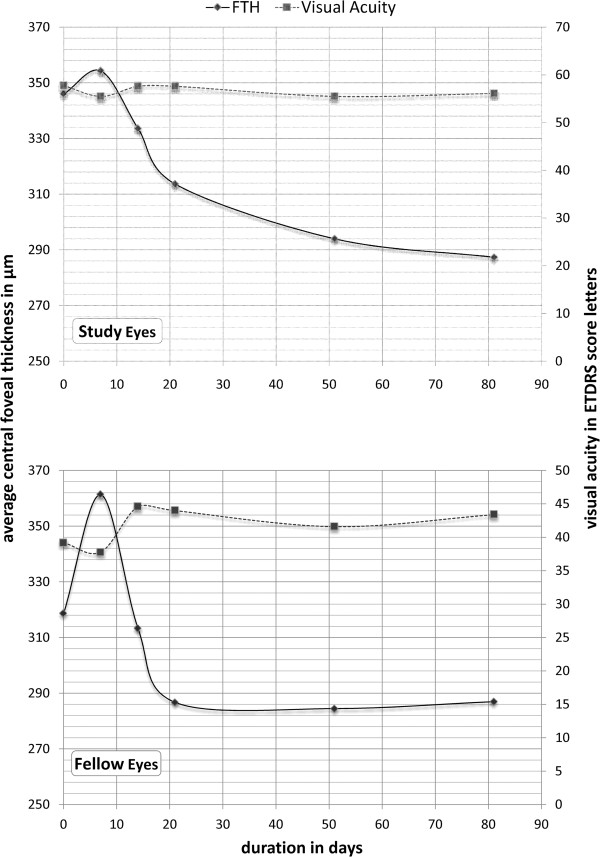
Mean changes in visual acuity and central retinal thickness during the study period in the study eyes (top) and the fellow eyes (bottom).

The mean foveal thickness of the central 1 mm of the retina (FTH) in the study eyes was 346 μm at baseline (Figure 3 top), with an excess FTH of 134 μm *[normal FTH = 212 μm as measured by OCT2 (Carl Zeiss Meditec, Inc.)].* After the 4th infusion *(end of treatment period)*, the mean FTH showed a 32.37 μm reduction (313.63 μm), representing 24.15% decrease in excess FTH. The change in FTH was not statistically significant when assessed using Wilcoxon signed rank test *(P = 0.674)*. FTH continued however to decrease during the follow-up period with thickness values of 294 μm and 287.38 μm, one and two months after the last treatment, respectively. The total reduction in FTH at the end of follow-up period (8 weeks after last infusion) represented 43.75% of the excess FTH at baseline *(p =0.161)*.

Despite the reduction in the central foveal thickness, BCVA remained mainly unchanged throughout the study. At baseline, mean BCVA in the study eyes was 57.75 letters (20/80-20/63). During the treatment period, the maximum mean BCVA was 57.63 letters, reached at week 3 *(before 3rd infusion)* and week 4 *(the end of treatment period, before 4th infusion,* i.e. *the patients only received 3 treatments at the time of the VA measurements)*, and the minimum was 55.5 letters reached at week 2 *(before 2nd infusion)*. During follow-up period, the mean BCVA was 55.5 letters at week 8 *(p =0.128)* and 56.13 (20/80) letters at week 12 *(p = 0.398)*, losing 1.63 letters when compared to the baseline visual acuity.

Fluorescein angiography images of the study eyes at baseline showed that six study eyes had macular leakage of 1 to 2 MPS disc areas. Two eyes had large CNV lesions with leakage of 10 to 12 MPS disc areas. Of these two eyes, one eye had extensive hemorrhage and the other eye showed disciform scarring. At the end of the treatment period (after 4th infusion), the FA images from five patients did not show any changes in the size of leakage area. Of the other three eyes, one eye showed mild reduction and two eyes showed mild increase in leakage.

#### Fellow eyes

Five of the fellow eyes had neovascular AMD with mean FTH of 318.67 μm at baseline (an excess FTH of 106.67 μm) (Figure 3 bottom). At the end of the treatment period (after 4th infusion), the mean FTH showed a 31.92 μm reduction (286.75 μm) representing 30% of excess FTH at baseline. The fellow eyes maintained the reduced FTH during the follow-up period with FTH of 284.5 μm and 287 μm, one and two months after the last infusion of CA4P, respectively. The change in foveal thickness was not statistically significant when assessed using non-parametric testing with Wilcoxon signed rank test with *p >0.5* at every visit.

At baseline, the mean BCVA in the fellow eyes was 39.2 ETDRS letters (20/160). At the end of the treatment period, there was a gain of 4.8 ETDRS letters (44 letters = 20/125). The fellow eyes maintained the improvement in BCVA. At the end of the follow-up period (8 weeks after last infusion), BCVA remained at 43.4 ETDRS letters (20/125). However, the improvement of BCVA was not statistically significant following third infusion (p = 0.39) and at the end of the study (p = 0.38).

## Discussion

Targeting the growth factors and signaling pathways involved in endothelial cell proliferation has led to the development and approval of the antiangiogenic therapies, pegaptanib and ranibizumab in neovascular AMD. The primary goal of these agents is to suppress the growth of new vessels underneath the retina. CA4P represents a lead compound in a separate group of agents known as VDAs that, unlike anti-VEGFs, are designed to selectively and rapidly compromise abnormal neovasculature. CA4P has been administered to patients with refractory solid tumors in multiple Phase 1, 2 and 3 trials [4,7,9,10].

Our study represents the first study of CA4P for an ophthalmic disease in humans. Clinical experience with CA4P consists of 18 completed and ongoing clinical trials in oncology and ophthalmology, together comprising more than 350 patients [4]. CA4P is currently being studied in patients with non-small cell lung cancer (NSCLC), anaplastic thyroid cancer (ATC) and platinum-resistant ovarian cancer. However, CA4P has not been investigated in patients without systemic malignancies until this study. As a VDA, it is expected that CA4P will have some vascular activities and that potential cardiovascular adverse events could be seen. In general, cardiovascular events have been observed at lesser frequency in doses of CA4P < 50 mg/m^2^. In our study, negative cardiac stress was an inclusion criterion; however, current studies with CA4P in oncology and ophthalmology do not require such testing as part of the screening procedures.

The class side effects of VDA encountered in oncology studies [7,9,10] were observed in our study population as well. However, CA4P was tolerated in our study without experiencing severe adverse effects of Grade-3 or 4 (CTCAE v.3.0). The most commonly encountered adverse event in our study was transient hypertension. Tubulin depolymerization is believed to be responsible of this transient elevation of blood pressure. Depolymerization of endothelial microtubules makes vessels more sensitive to vasoconstriction and, consequently, hypertension [4]. Elevation of blood pressure is of particular concern in our study population giving the susceptibility and high prevalence of hypertension in the age group of neovascular AMD. The majority of our study patients had either a pre-existing or a borderline hypertension, which, along with other factors such as the presence of higher risk factors of cardiovascular disease in our study’s age group, may be responsible for the higher incidence of post-infusion hypertension in our study (75%) when compared with the reported rates in oncology (30%) [11]. In support of our explanation, the SANA study [12] have demonstrated that when bevacizumab was administered intravenously in patients with neovascular AMD it resulted in an incidence of hypertension (78%), which is comparable to our study results. The BEAT-AMD Study has reported a mean elevation of systolic blood pressure from 140 mmHg to 150 mmHg in patients who received bevacizumab intravenously [13]. In another study, Geitzenauer et al. reported comparable (to ours) elevation in blood pressure following intravenous administration of bevacizumab in patients with neovascular AMD, which has peaked in the second day post infusion. In all previous studies that evaluated intravenous bevacizumab, the investigators have concluded that the elevated blood pressure is insignificant [14]. However, despite having generally comparable results, our study cannot be directly compared with the previous studies due to the different methodologies employed and different patient characteristics. For example, it is not known from the BEAT-AMD Study, whether blood pressure has or has not elevated in the immediate post-infusion hours, which was when we noticed the transient hypertension in our patients [13]. In addition, while our study has allowed patients with high blood pressure to participate, Geitzenauer et al., have excluded patients with blood pressure >140/90 mmHg and patients taking more than one drug to control their blood pressure; they also did not allow bevacizumab infusion if pretreatment blood pressure is >140/90 mmHg [14]. Therefore, our results should be taken within their own context and any comparison with previous studies that utilized other intravenous agents should be interpreted with caution.

Angiotensin converting enzyme (ACE) inhibitors were used to manage the elevated blood pressure in our study. However, experiences from more recent studies suggest that management guidelines using nitrates and calcium channel blockers can result in a decrease in cardiovascular toxicity [4,15]. Giving the short-lived duration of elevated pressure, which can be explained by the short plasma half-life of CA4P, the control of hypertension may only be required for few hours following the infusion. Routine prophylaxis with a calcium channel blocker could also become part of the treatment in high-risk patients [4].

Similar to the elevation in blood pressure, the changes in QTc were generally mild and confined to the post-infusion period. QTc could not be predicted from baseline values. Current clinical trials, in both oncology and ophthalmology, implement guidelines with magnesium and potassium supplementation to decrease or prevent episodes of QTc prolongation.

Consistent with previous studies [8,16,17], intravenous CA4P did not show any cytotoxic side effects, and also did not demonstrate adverse effects previously reported with intravenous anti-VEGF agents, such as proteinuria, hemorrhage or thrombosis; however, our pilot study sample was too small to uncover all potential AEs associated with CA4P.

There were no serious ocular AEs associated with CA4P therapy. Such result should be interpreted with caution giving the small sample size and the non-randomized open label design of this pilot study.

The observations in our study further extend the reported effects of CA4P in animal models of ocular disease [5,6] with suggestive evidence of biological activity in human subjects with neovascular AMD. Such effect is evidenced by the reduction of the excess foveal thickness in the study eyes by 24.15% and 43.75% at the end of the treatment and follow-up periods, respectively. A similar sustainable reduction was also observed at the end of the treatment period in the fellow eyes that had CNV. The results need to be taken with caution giving the limitations of the OCT technology utilized in our study. Other studies utilizing the newly emergent spectral domain technology may shed different light on the anatomical outcome of CA4P therapy in neovascular AMD. The fact that the FTH reduction in our study is much less pronounced than with anti-VEGF as demonstrated in some studies [12,14] can be explained in part by the advanced disease of our study sample. It is also possible that a vascular disrupting agent does not have as significant effect in reducing retinal edema as an anti-VEGF agent.

At the functional level, BCVA remained stable in the study eye for the 12-week duration of the study, which may be attributed to either the nature of the disease or the bioactivity of CA4P. As the number of study subjects was small, it would not be appropriate to generate conclusions regarding the bioactivity of the drug. However, the visual gain in the fellow eye and the stability of BCVA in the study eye, in addition to the observed reduction of the retinal thickness in both study and fellow eyes, may warrant further exploration of the potential beneficial effects of CA4P in eyes with neovascular AMD. Significant visual gain has been reported in patients who received intravenous bevacizumab [12,14]. In the SANA study patients gained a median of 8 letters over 12 weeks [12]. Nevertheless, all studies were non-controlled, non-randomized, open-labeled, and small sampled; hence, no directed comparison can be accurately drawn between both studies. In the only controlled study, the BEAT-AMD Study did not demonstrate significant change in BCVA in patients with neovascular AMD when treated with intravenous bevacizumab, which seems consistent with our results [13]. All patients enrolled in our study presented at baseline with active disease despite at least two sessions of PDT, which perhaps indicate a level of severity that may not be present in many of the anti-VEGF studies.

No maximal tolerated dose (MTD) was determined in this study. The dose levels employed, 27 to 36 mg/m^2^, are below the MTD (approximately 60 mg/m^2^) that was independently determined in three separate oncology studies [9,10]. The dose level of 27 mg/m^2 ^was approximately the threshold dose for inhibition of blood flow in oncology studies. Investigators concluded that significant impact on tumor blood flow was observed in a higher proportion of patients when dose levels of 40 to 60 mg/m^2^ were administered [4,7,9,18]. Further assessment of the dose dependency of CA4P for CNV might be of interest due to the potential for greater biological effect with higher doses, provided no additional or more severe adverse events are noted.

Compared with the current intraocular treatment options that are available for patients with neovascular AMD, agents administered intravenously, especially those with potential systemic effects, are admittedly less appealing to patients and ophthalmologists. However, the novelty of mechanisms though which CA4P exerts its biological activity may add to the expanding arsenal of therapeutic options available today for patients with neovascular AMD. Furthermore, recent studies in rabbits and primates have suggested that topical administration of CA4P allows sufficient penetration of the drug to the choroid [19]; hence, localized ocular therapy with CA4P may be feasible and available in the future. In addition, further study of systemically administered CA4P in AMD may be warranted, especially if the associated side effects are known and can be controlled with proper therapy and monitoring.

Although CA4P is an anti-vascular agent, its mechanism of action and side effect profile differs from that of anti-angiogenic VEGF inhibitors. Synergetic inhibitory effects of CA4P and bevacizumab on blood flow have been demonstrated in recent studies in both xenograft models and in patients with refractory solid tumors. CA4P may, therefore, have potential role in the management of neovascular AMD and other retinal vascular diseases, either as monotherapy or in combination with anti-VEGF treatments, especially if topical or intravitreal or other local formulation/s are developed.

## Conclusion

The safety profile of intravenous CA4P was consistent with that reported in oncology patients and with the class effects of vascular disruptive agents. There are evidences to suggest efficacy of CA4P in neovascular AMD. However, the level of systemic safety and efficacy indicates that systemic CA4P may not be suitable as an alternative monotherapy for neovascular AMD, especially when compared to the overall safe and very effective intravitreal anti-VEGF therapy today. There might be a role for CA4P as an adjunctive therapy, to be used in combination approach, when delivered intravitreally/topically. Further studies of CA4P in AMD and other ophthalmic disorders are indicated to investigate the potential role of vascular disrupting agents in the management of angiogenic retinal vascular diseases.

## Competing interests

Jai Balkissoon is the Vice President, Clinical Research and Clinical Operations, OXIGENE, Inc.

## Authors’ contributions

MI has contributed in data analysis and interpretation, have been involved in drafting and critical revision of the manuscript, and have given final approval of the version to be published; DVD has contributed in the concept and design of this study, have been involved in data interpretation and in critical revision of the manuscript, and have given final approval of the version to be published; YJS has contributed in data analysis and interpretation, have been involved in drafting and critical revision of the manuscript and have given final approval to this version to be published; SMS has contributed in the concept and design of this study, have been involved in data collection and critical revision of the manuscript, and have given final approval of the version to be published; EVA has contributed in data collection and critical revision of the manuscript and have given final approval to of the version to be published; GH has contributed in data collection and critical revision of the manuscript and have given final approval to of the version to be published; JKD has contributed in data analysis and interpretation, have been involved in data collection and in drafting and critical revision of the manuscript, and have given final approval to of the version to be published; RR has contributed in data analysis and interpretation, have been involved in data collection and in drafting and critical revision of the manuscript, and have given final approval to of the version to be published; JB has contributed in the concept and design of this study, have been involved in critical revision of the manuscript, and have given final approval of the version to be published, JTH has contributed in the concept and design of this study, have been involved in data collection and critical revision of the manuscript, and have given final approval of the version to be published; PAC has contributed in the concept and design of this study, have been involved in data collection and critical revision of the manuscript, and have given final approval of the version to be published; QDN has contributed in the concept and design of this study, has been involved in data collection and critical revision of the manuscript, and has given final approval of the version to be published. All authors read and approved the final manuscript.

## Pre-publication history

The pre-publication history for this paper can be accessed here:

http://www.biomedcentral.com/2050-6511/14/7/prepub
